# A predictive machine learning model for cannabinoid effect based on image detection of reactive oxygen species in microglia

**DOI:** 10.1371/journal.pone.0320219

**Published:** 2025-03-25

**Authors:** Patricia Sinclair, William Jeffries, Nadege Lebert, Maheen Saeed, Aman Ullah, Nadine Kabbani

**Affiliations:** 1 Interdiscplinary Program in Neuroscience, George Mason University, Fairfax, Virginia, United Sates of America; 2 Bioinformatics and Computational Biology, George Mason University, Manassas, Virginia, United Sates of America; 3 School of Systems Biology, George Mason University, Manassas, Virginia, United Sates of America; University of Texas Medical Branch at Galveston, UNITED STATES OF AMERICA

## Abstract

Neuroinflammation is a key feature of human neurodisease including neuropathy and neurodegenerative disease and is driven by the activation microglia, immune cells of the nervous system. During activation microglia release pro-inflammatory cytokines as well as reactive oxygen species (ROS) that can drive local neuronal and glial damage. Phytocannabinoids are an important class of naturally occurring compounds found in the cannabis plant (*Cannabis sativa*) that interact with the body’s endocannabinoid receptor system. Cannabidiol (CBD) is a prototype phytocannabinoid with anti-inflammatory properties observed in cells and animal models. We measured ROS in human microglia (HMC3) cells using CellROX, a fluorescent dynamic ROS indicator. We tested the effect of CBD on ROS level in the presence of three known immune activators: lipopolysaccharide (LPS), amyloid beta (A*β*_42_), and human immunodeficiency virus (HIV) glycoprotein (GP120). Confocal microscopy images within microglia were coupled to a deep learning model using a convolutional neural network (CNN) to predict ROS responses. Our study demonstrates a deep learning platform that can be used in the assessment of CBD effect in immune cells using ROS image measure.

## Introduction

Cannabidiol (CBD) is a non-psychoactive phytocannabinoid found in the cannabis plant and is one of over 100 identified cannabinoids [1]. Preclinical studies have shown that CBD suppresses pro-inflammatory cytokines, such as IL-1β, IL-6, and tissue necrosis factor α (TNF-α), *in vitro* and *in vivo* experimental models of inflammation, cancer, and neurodegenerative disease [2–4]. Additionally, both preclinical and clinical research has highlighted CBD’s therapeutic potential across a range of human disorders, including those associated with high cellular oxidative stress, a state marked by an imbalance in free radicals within the body. Studies indicate that CBD can inhibit oxidative stress in retinal neurons of diabetic animals, reducing glutamate accumulation and preventing neuronal cell death [5–7]. CBD’s role in modulating redox balance by altering the levels and activities of both oxidants and antioxidants has garnered significant clinical interest, particularly for its potential in managing neuroinflammatory conditions [8].

Microglia are the primary immune cells of the central nervous system, displaying rapid functional plasticity in response to environmental stimuli [9]. Altered microglial function is a hallmark of several neurodegenerative diseases, including Alzheimer’s [10]. Microglial transitions between (M1 and M2) functional states are accompanied by a metabolic switch to meet the energy demands of the cell [11,12]. For instance, pro-inflammatory (M1-like) microglia primarily rely on glycolysis, de novo fatty acid synthesis, and the pentose phosphate pathway, while anti-inflammatory (M2-like) microglia depend on oxidative phosphorylation (OXPHOS) to facilitate tissue repair [12]. These metabolic shifts directly impact the production of cellular reactive oxygen species (ROS), which serves as an indicator of the inflammatory status of the microglia [13].

ROS plays an important role in the progression of diseases, including inflammation, atherosclerosis, aging, and age-related degenerative disorders [10]. Various types of ROS, including superoxide anions (O2^−^) and hydrogen peroxide (H_2_O_2_), are produced as byproducts of cellular metabolism [14]. Dynamic measures of ROS are also crucial for evaluating the toxicological effects of bioactive compounds [15,16]. Phytocannabinoids, especially CBD, have been shown to directly reduce ROS levels, contributing to neuroprotection and anti-inflammatory effects in conditions such as Parkinson’s disease [3,8]. The detection and analysis of ROS using dynamic fluorescent dyes such as CellROX have contributed to the analysis of free radical management and oxidative stress in neural cells [14]. Coupled with high resolution confocal microscopy and machine learning, new tools are being developed for assessing the effects of CBD. In this study, we developed a predictive image detection platform capable of distinguishing between ROS responses in the presence of CBD and several known immune activating compounds in human microglial cells. This platform presents a novel tool for testing CBD mitigation of oxidative stress in microglia.

## Methods

### Cell culture and reagents

Human microglial HMC3 cells (ATCC CRL-3304) were propagated in T75 flasks with DMEM (Gibco 11995065) supplemented with 10% fetal bovine serum (FBS) and 1% pen/strep and incubated (37°C with 5% CO_2_). Transfected cells were plated onto 24-well glass-bottom plates coated with 100 μg/ml poly-D-Lysine (Millipore A-003-E) and allowed to adhere overnight prior to applying the HIV-protein GP120 (500 pM, catalog #12582), A*β*_42_ (100 nM, Bachem 4045866), or lipopolysaccharide (LPS 100 ng/ml, Millipore Sigma #L2630) [17], with or without pretreating for 12 hours with cannabidiol dissolved in DMSO and diluted in media (CBD 10 μg/ml, Phytolabs #PHL85705). An equal volume of DMSO was applied to control cells not treated with CBD. The HIV-GP120 protein was obtained from the National Institutes of Health (NIH) AIDS Reagent Program. All experiments were conducted on cells not exceeding 20 passages.

### DNA transfection and cellrox detection

A pcDNA3.1 DNA plasmid encoding Lifeact-mCherry (Addgene plasmid #67302) was propagated in DH5*α* competent cells (ThermoFisher #18258012) and isolated with the Qiagen maxi-prep kit (#121612). HMC3 cells were transfected using Lipofectamine 2000 (ThermoFisher, 11668030) per the manufacturer instructions. Briefly, cells were incubated (37°C/5% CO2) with Lipofectamine 2000 reagent and the plasmid in serum-free media for 6 hours before seeding on a PDL coated 24-well glass bottom plate in full-serum media. In some experiments, cells were pre-treated with CBD or vehicle for 12 hours before drug challenge. At the end of 24-hours, treatment media was exchanged for media containing 5 μM CellROX green (ThermoFisher #C10444) and incubated for 30 minutes according to the manufacturer instructions. CellRox was visualized in cells fixed with 3.7% paraformaldehyde for 15 minutes at room temperature while in some experiments, CellROX was also visualized live in cells. Z-stack images (0.5μm/section) were collected using confocal microscopy within 24 hours on a Zeiss LSM 800, with 488 nm/520 nm excitation/emission (for CellROX) and ex/em 555 nm/620 nm (for Lifeact-mCherryactin) filters, using the Zen software package (Carl Zeiss AG, Oberkochen, Germany). Individual z-stack images slices were saved as TIFF files for machine learning and.czi files for quantitative analysis.

### CellROX quantification

Three-dimensional (3D) reconstructions of individual cells were created based on total Lifeact-mCherry cell signal. Total ROS within each cell was measured using FIJI/ImageJ analysis of the CellROX signal [18]. Statistical differences between the averaged cellular CellROX fluorescent signals was determined between challenge-vehicle or challenge-CBD treated experimental groups using Welch’s two-tailed t-tests in R-statistical software [19–21]. p-values <  0.05 were considered statistically significant. Bar graphs were created using ggplot in the R-studio environment [19].

### Deep-learning dataset

The imaging dataset was divided into six groups based on the cell challenge condition: LPS, GP120, or Amyloid (A*β*_42_) and pretreatment condition: CBD or control (DMSO). Each 3D cell image consisted of 18–20 individual 1024 x 1024-pixel 2D z-stack slices saved as high resolution TIFF images that was usedto create the dataset for each group.

### Image pre-processing

The data augmentation strategy in this study consisted of using individual 2D z-stack slices rather than the combined 3D cell image sample. This provided a twenty-fold increase in the amount of image data available for machine learning (Table 1). To account for image variability, pixel values were normalized using Equation 1, converted to grayscale, and set to reduced dimensions (256 pixels x 256 pixels). Image contrast was enhanced using histogram equalization in OpenCV Python library [22] to maximize the intensity range of pixel values Equation 1:

**Table 1:** Dataset by condition and treatment.

Condition	Treatment	Raw 3D Images	Processed 2D Images
LPS	CBD	124	484
LPS	DMSO	130	471
GP120	CBD	66	403
GP120	DMSO	118	608
A*β*_42_	CBD	122	538
A*β*_42_	DMSO	138	735


$$value_{new�}=�\frac{\left(value_{old}�-�value_{min}\right)}{\left(value_{max}�-�value_{min}\right)}\cdot 255$$

### Deep learning models

We used 72/8/20 2D (TIFF) images for training, validation, and testing, respectively. Cross entropy loss was used as the loss function and optimized with the Adam algorithm [23]; the learning rate was 0.0001. All deep learning components were implemented using PyTorch [24]. The condition model was used to classify CellROX images as having been conditioned with LPS, GP120, or A*β*_42_. Its architecture is the same as the treatment model but using a 3-class classification at the Softmax layer.

## Results

### Detection and analysis of ROS in human microglia

**Fig 1:**
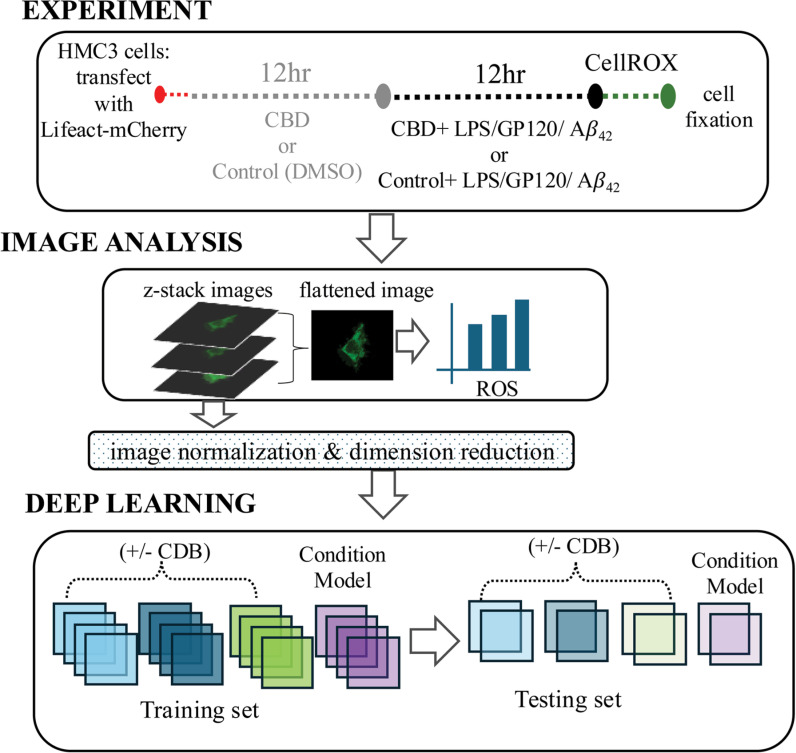
Schematic of the experimental design. HMC3 microglia cells were transfected with Lifeact-mCherry treated with CBD or vehicle followed by LPS, GP120, or A*β*_42_. Confocal imaging within CellROX labeled cells was used for machine learning as well as analysis. A deep learning model was trained using z-stack slices to predict the CBD and the specific treatment condition.

Cellular ROS level correlates with the inflammatory status of immune cells [3]. We examined the effect of CBD on CellROX within HMC3 microglial cells in the presence of known immune chemical challenges: LPS, A*β*_42_, and the HIV glycoprotein GP120. As shown in the workflow summary (Fig 1), HMC3 microglia were transfected with Lifeact-mCherry (actin) and then treated with 10ug/mL CBD or the vehicle (DMSO) as the control condition. The immune challenge, 100ng/mL LPS, 100nM A*β*_42_, or 500pM GP120, was then presented in the presence of CBD or the control. At 30 min prior to the end of the experiment, CellROX was loaded into living cells for the detection of ROS [25]. Imaging analysis of was conducted using confocal microscopy of transfected actin and CellROX within the same cell across all experimental conditions. Individual images were collected in 0.5μm z-stack sections and used to train, validate, and test deep-learning prediction models across the treatment conditions (Fig 1). The total number of z-stack sections obtained per condition is summarized in Table 1.

**Fig 2:**
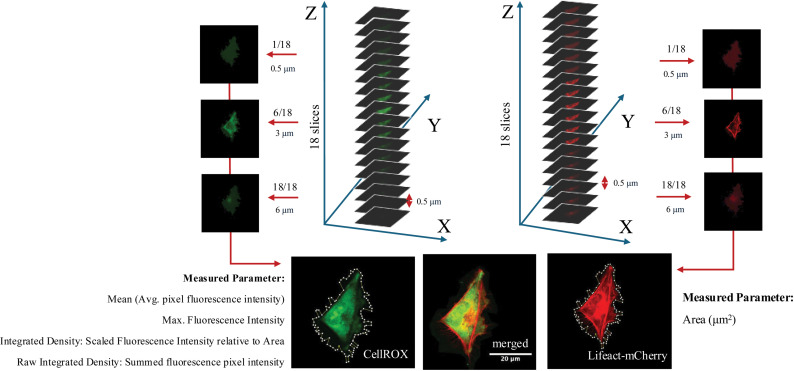
Components of the cell image analysis. The Lifeact-mCherry signal (red) was used to outline individual cells and to measure their surface area (μm^2^). The CellROX signal (green) was used to measure average pixel fluorescence intensity; maximal pixel fluorescence intensity; the integrated density relative to the surface area; the raw integrated density (sum of pixel fluorescence intensity).

Various species of ROS exist in cells and are modified by the cellular environment [14]. We confirmed the effect of LPS on increased ROS in HMC3 microglia using CellROX imaging (S1 Fig). CBD has been shown to reduce ROS levels within microglial cells, under various conditions, thereby contributing to anti-inflammatory and neuroprotective effects [26]. Confocal imaging was used to obtain information on the spatial distribution and relative signal intensity of CellROX within microglia. As shown in Fig 2, the CellROX signal was seen throughout the cell showing a varied punctate distribution consistent with ROS production in various organelles including the nucleus [27]. X, Y, and Z plane analysis of confocal stack sections were used to measure a3D CellROX signature pattern in cells across eachexperimental condition. Actin co-labeling was used to define the edges and contour of the cell and to obtain a measure of surface area. Values corresponding to the CellROX signal and surface area were analyzed in Image J to obtain average mean and maximum signal intensity as well as raw and integrated density values across the experimental conditions.

### Effect of CBD on LPS, A*β*_42_, and GP120 associated ROS level

**Fig 3:**
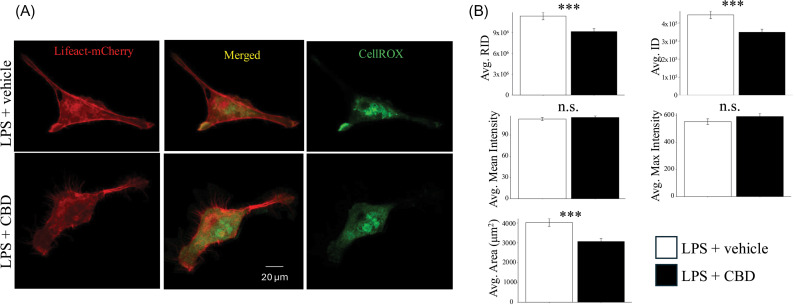
The effect of CBD in LPS treated microglia. A. Representative images of the ROS signal in LPS and CBD +  LPS treated HMC3 cells. B. Average ROS signal in LPS and CBD +  LPS treated cells. (*: p <  0.05, **: p <  0.01, ***: p <  0.001).

LPS is a bacterial endotoxin known to drive inflammatory responses in microglia leading to increased ROS levels, cytokine production, and DNA damage [28]. We determined the effect of CBD on LPS-mediated changes in cellular ROS. Pre-treatment with CBD was found to significantly attenuate the CellROX signal within HMC3 cells. Specifically, CBD exposure was associated with a significant reduction in CellROX mean, raw integrated density, integrated density, and area of HMC3 cells treated with LPS (n =  84-88 cells, p(RID) =  1.27x10^-3^, p(ID) =  4.61x10^-4^, p(Max) =  0.22, p(Mean) =  0.47, p(Area) =  1.21x10^-4^) (Fig 3).

**Fig 4:**
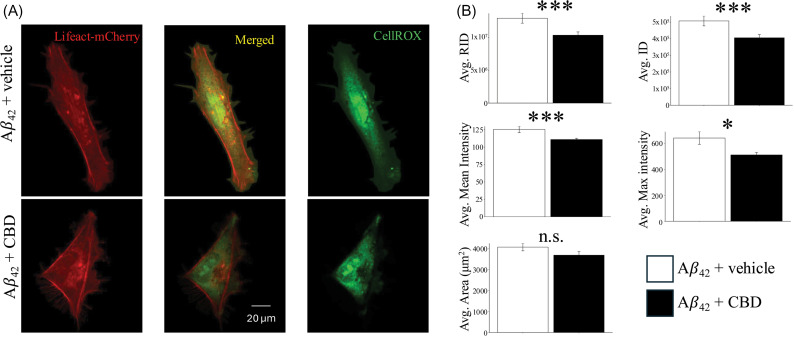
The effect of CBD in A*β*_42_ treated microglia. A. Representative images of ROS in A*β*_2_ and CBD +  A*β*_42_ treated HMC3 cells. B. Average ROS signal in A*β*_42_ and CBD +  A*β*_42_ treated cells. (*: p <  0.05, **: p <  0.01, ***: p <  0.001).

We tested the effect of CBD on ROS levels within microglia treated with the cytotoxic amyloid peptide A*β*_42_. Previous studies have shown the A*β*_42_ exposure increases the inflammatory properties of microglia leading to elevated ROS and a disruption to the mitogen signaling pathway [29]. Our findings show that treatment with CBD significantly decreases the effect of A*β*_42_ on the CellROX signal including mean, raw integrated density, integrated density, intensity, and maximum intensity (n =  103-119 cells, p(RID) =  4.73x10^-3^, p(ID) =  4.73x10^-3^, p(Max) =  0.014, p(Mean) =  3.46x10^-3^, p(Area) = .13) (Fig 4).

**Fig 5:**
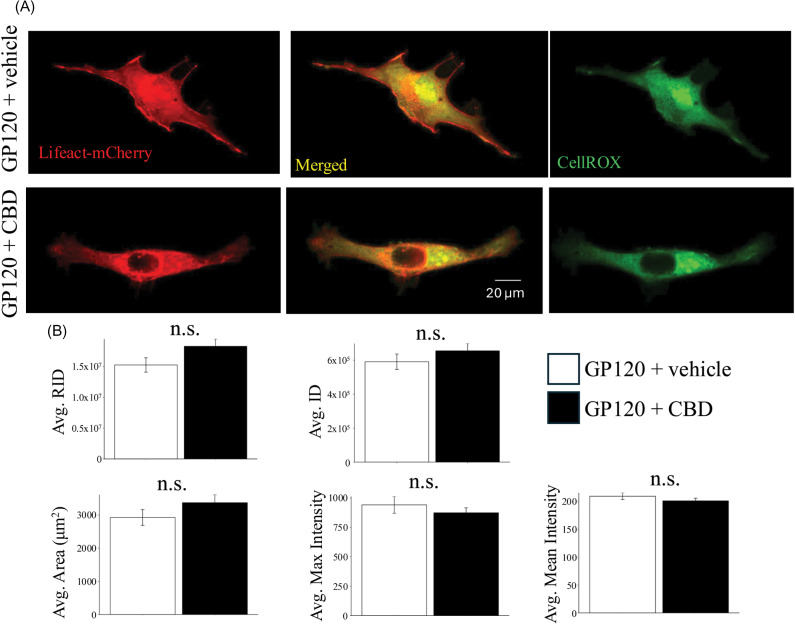
The effect of CBD in GP120 treated microglia. A. Representative images of ROS in GP120 and CBD +  GP120 treated HMC3 cells. B. Average ROS signal in GP120 and CBD +  GP120 treated cells. (*: p <  0.05, **: p <  0.01, ***: p <  0.001).

A similar analysis was conducted to determine the effect of CBD on ROS levels in the presence of the HIV glycoprotein GP120 that has been implicated in the neuroinflammatory pathology of HIV associated neurodegenerative disease (HAND) [30]. Previous studies conducted in microglia show that GP120 increases the production of ROS from mitochondria [31]. Our analysis with CellROX shows that CBD does not significantly alter ROS levels within HMC3 cells treated with GP120. Statistical analysis (n =  44-49 cells, and p(RID) =  0.42, p(ID) =  0.09, p(Max) =  0.97, p(Mean) =  0.81, p(Area) =  0.19) (Fig 5).

### Development of predictive deep learning models based on ROS responses

**Fig 6:**
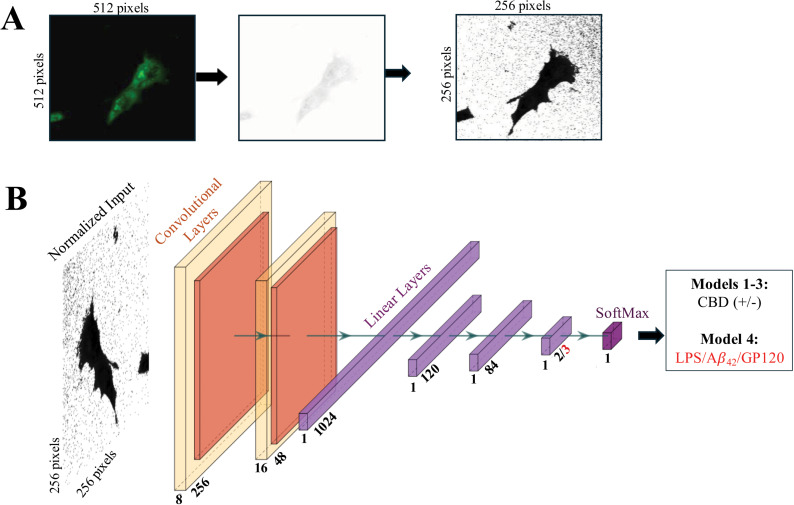
Image processing and the development of the deep learning models. A, raw z-stack slice image of the ROS signal (left) is normalized (middle) and then transformed using histogram equalization (right). B, the architecture of a convolutional neural network (CNN) used to train the model by passing images through two convolutional layers, three linear layers, and one Softmax layer. Models 1-3 (black) were developed to predict the presence (+) or absence (−) of CBD in the experiment, Model 4 (red) was developed to distinguish between LPS, A*β*_42_, or GP120.

Convolutional neural networks (CNN) have been used extensively in image classification tasks [32]. Convolutional layers extract features by using filter maps to scan all regions of an image for patterns. The method is invariant to rotation of the image, adding to the method’s success and popularity in the field of image classification. We used a deep learning strategy to develop a predictive model for ROS responses to CBD treatment in microglial. Deep learning models typically require a large amount of data to learn how to perform a given task with various data augmentation mechanisms applied. Our model used training/validation/testing splits of the pre-processed data obtained from 2D sections (z-stack) images of the CellROX signal. Image pre-processing was conducted as described in Methods. Briefly, the fluorescent CellROX image was converted to grayscale then normalized (Fig 6A). A deep learning model was built on reduced image complexity and generalizability amongst the fluorescent images.

We developed 3 predictive models (Models 1-3) that can classify images based on the presence of CBD or the control (DMSO) condition. The models use two convolutional layers and three linear layers to classify CellROX images as being treated with CBD (positive) or control (negative). The first convolutional layer processes a 256x256 pixel image using eight filter maps with a kernel size equal to 16. A 5x5 max pooling layer is applied to a 0.20 dropout layer to prevent overfitting. The input to the second convolutional layer is 8x48x48 and uses 16 filter maps with a kernel size of 5. Successive dense linear layers were then used to reduce the input from 1x1024 down to 1x2. The final 2-class categorization that predicts of the presence or absence of CBD (+/-) was computed using Softmax software (Fig 6B**, black**).

**Fig 7:**
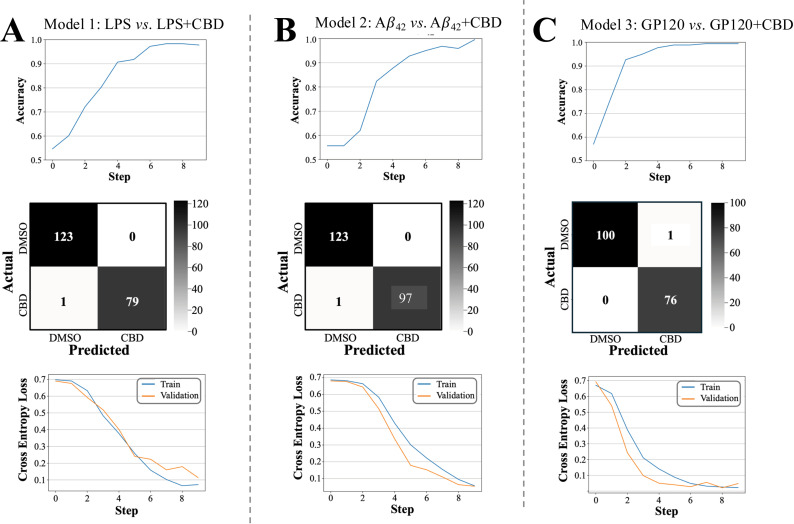
Performance analysis of models 1-3. Top panels: Accuracy curves display the model progression over ten epochs and measure the percentage of test cases correctly predicted. Middle panels: Confusion matrices show the number of true negatives (upper left), false positives (upper right), false negatives (lower left), and true positives (bottom right). Bottom panels: Cross-entropy training and validation loss curves show that all models are not overfitted.

Models 1-3 were trained using image data on LPS, GP120, and A*β*_42_ using a common architecture. Model performance results for each group is presented in Fig 7 as accuracy curves, confusion matrices, and loss curves. Receiver-operator-characteristic (ROC) are shown inS2 Fig. Model accuracy was used to measure model performance based on its ability to correctly classify the image as CBD (+) or control (−). Results show that all three models achieved >  90% accuracy by the fifth epoch while all models achieved >  99% accuracy by the tenth epoch. Confusion matrices were generated to examine the occurrence of false positive and false negative outcomes. These matrices show high predictive model performance. Lastly, we used a cross-entropy loss method, which measures loss on a 0–1 scale with 0 reflecting perfect model performance. As indicated in Fig 7, convergence of the training and validation loss curves suggests little to no overfitting within our models.

### Condition deep learning model training and performance

**Fig 8:**
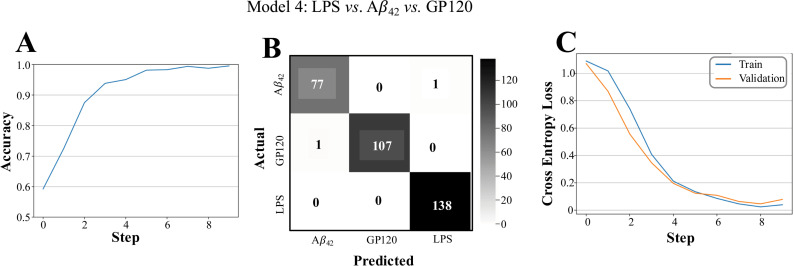
Model 4 performance analysis. A, Accuracy curves display the model progression over ten epochs and measure the percentage of test cases correctly predicted. B, Confusion matrices show the number of true negatives (upper left), false positives (upper right), false negatives (lower left), and true positives (bottom right). C, Cross-entropy training and validation loss curves show that all models are not overfitted.

While the above models use a 2-class categorization to predict conditions that distinguish the presence or absence of CBD across the three challenge conditions (LPS, GP120, and A*β*_42_), we sought to develop a model that can distinguish between the three challenge conditions. This “condition model” (Model 4) was developed using the same settings as earlier however using the stimulating factor (i.e., LPS, GP120, and A*β*_42_) only training set. In this case, a 5x5 max pooling layer was applied to a 0.20 dropout layer to prevent overfitting. The input to the second convolutional layer was 8x48x48 using 16 filter maps with a kernel size equal to 5. Successive dense linear layers where then used to reduce the input from 1x1024 down to 1x3 and the final network ternary prediction computed using Softmax software (Fig 6B**, red**). Model 4 was found to achieve strong performance scores as determined by accuracy curve, confusion matrix, and loss curves computations (Fig 8). The robust performance of the various models supports the ability of CNN to predict differences in ROS image signals within HMC3 cells.

### Model interpretability

**Fig 9:**
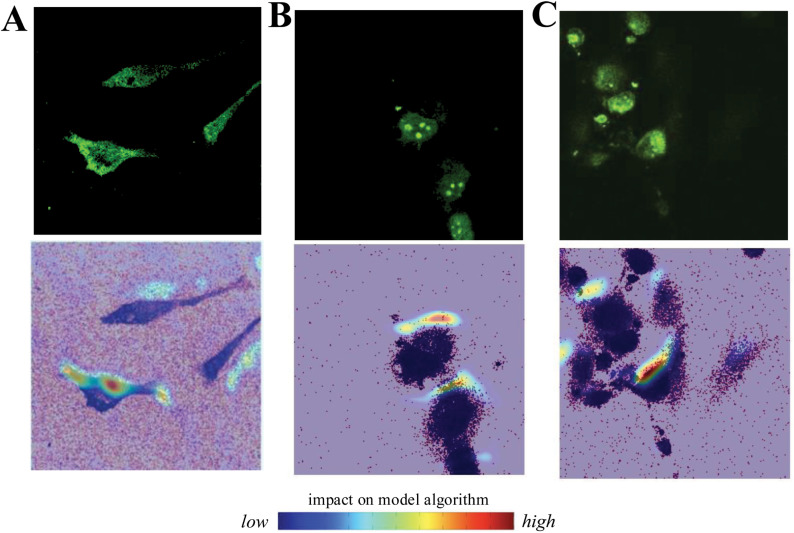
Saliency maps for CBD treated cells. A comparison of ROS fluorescent images (top) and saliency maps (bottom) between CBD treated cells that were stimulated with LPS (A), GP120 (B), and A*β*_42_ (C).

One of the main challenges with deep learning models is interpreting what the model is learning. Neural network architectures, with their multiple layers and nonlinear attributes, act like black boxes wherein only the output can be understood and used [33]. There has been increasing effort to develop new tools to aid in model interpretability for CNN models [34,35]. Gradient-weighted Class Activation Mapping (Grad-CAM) [36], uses the learned weights of the convolutional layers to generate saliency maps that highlight important regions within the image used for classification. To interpret our CBD treatment predictive models, we implemented Grad-CAM and passed samples of LPS, GP120, and A*β*_42_ treated cells through their respective learned models. As shown in Fig 9, representative Grad-CAM saliency maps are generated for each of the three CBD treatment model conditions. It is interesting to note that all three images indicate high saliency at the cellular edge. While these saliency maps do not quantify attributes, they may be useful to inform biological hypotheses on the effects CBD on cellular ROS.

## Discussion

CBD is a non-psychotropic phytocannabinoid with neuromodulatory and neuroprotective properties in various animal models including the 6-hydroxydopamine (6-OHDA), 1-methyl-4-phenyl-1,2,3,6-tetrahydropyridine (MPTP) rodent model of Parkinson’s disease [3,8]. Neurodegenerative diseases often involve microglial activation and elevated ROS in affected brain regions [37]. High levels of ROS can cause oxidative stress, leading to neuronal death, exacerbated neuroinflammatory signaling, and synaptic damage. In this study we assessed the effect of CBD, a promising therapeutic compound for the treatment of inflammation, using ROS responses in human microglial HMC3 cells. We tested the ability of CBD to attenuate ROS when co-applied with 3 known immune activators: LPS, A*β*_42_, and GP120. LPS is a known activator of toll-like receptor (TLR)-4 and nuclear factor kB (NF-κB) pathways in microglial cells [38]. At similar concentrations to those used in our study, LPS stimulation has been shown to alter microglial morphology, density, and the expression of phagocytic markers as well as increase cellular ROS [39].

We confirmed the effect of LPS on ROS signal enhancement and found that CBD application significantly reduced ROS levels. This finding supports the anti-inflammatory properties of CBD and pairs the ROS signal with the inflammatory status in HMC3 cells. We conducted similar analyses of ROS in response to the presentation of neurotoxic peptides A*β*_42_ and GP120. These two peptides have been shown to activate microglia increasing ROS production in the mitochondria [40]. Our experiments show that CBD attenuates A*β*_42_ associated ROS production however we did not detect an effect of CBD on GP120 associated ROS responses within HMC3 cells. Based on this result, it is plausible that the ability of CBD to reduce ROS within microglia is dependent on the source of the chemical stimulator.

How CBD mitigates inflammatory signaling within immune cells, such as microglia, is not understood. Studies show that CBD has various molecular targets in addition to its relatively low affinity for cannabinoid CB1 and CB2 receptors that are expressed in immune cells [41]. CBD is also shown to bind GPR55 receptors, transient receptor potential vanilloid (TRPV1/2) and serotonin 5-HT1A receptors [42–44]. CBD’s lipophilic properties also enable it to translocate across the plasma membrane, and CBD has been shown to bind CB receptors as well as the voltage dependent anion channel (VDAC) within mitochondria [45]. Interestingly, autooxidation via the mitochondrial respiratory chain is a chief source of cellular ROS, and mitochondria represent an important interface for CBD modulation of ROS in microglia. Changes in ROS levels have been shown to promote the release of ROS from cells contributing to damage of nearby neurons as well as glia through lipid peroxidation and the oxidation of amino acids in various proteins [14]. Monitoring ROS is an important strategy for the assessment of immune cell activation.

By coupling CellROX imaging to deep learning using CNN we developed 4 models that can classify images of microglia, with high accuracy, based on cellular ROS signatures for the presence or absence of CBD. Our models employ standard machine learning best practices to ensure stronger performance while aiming to reduce overfitting. These best practices included data augmentation, training/validation/testing splits, and the injection of random noise in the form of convolutional dropout layers. As a result, all three CBD prediction models achieved over 99% accuracy on holdout test sets and showed no signs of overfitting by inspection of the loss curves. Loss is a machine learning term to indicate the degree in which a model is inaccurate during its training cycles. In our model, we use Cross Entropy Loss which measures loss on a 0–1 scale, where 0 equals a perfect model. If overfitting had occurred, clear divergence would be seen in the loss curves between the train and validation curves. In fact, training and validation loss curves showed the same trend within each of the models, and confusion matrix findings were limited due to the small number of misclassified samples and no pattern in false positives and negatives can be discerned. The results of our 4 models suggest that this machine learning approach may be generalized to future images generated outside of the current training exercise and may be of value in assessing the effectiveness of CBD as a ROS reducing agent in immune cells. To further solidify this CBD diagnostic tool, future work should test the models against ROS images produced outside of this study, including in human induced pluripotent stem cell (iPSC) derived microglia or macrophages.

At this stage, ROS image results between the experimental findings and the deep learning models show overall robustness amongst the three activation conditions (LPS, A*β*_42_, and GP120). Interestingly, imaging results indicate that CBD did not have a significant effect on GP120 associated ROS expression within microglia (Fig 5), yet the deep learning model appears able to identify GP120 images within the dataset. It is plausible that the deep learning model can identify image features including signal patterns not directly measured by our experimental analysis. Intriguingly, saliency map analysis suggests the importance of measures at the cellular edge while experimental analysis involves quantifying the fluorescent ROS signal throughout the cell. These discrepancies speak to the potential for bias within the experimental analysis and suggest a role for machine learning in improving the assessment of ROS. While it is not clear if/how these saliency maps relate to an effect of CBD on ROS levels and localization within the cell, it is known that ROS production and release occurs through enzymatic activity near the plasma membrane and involves NADPH oxidase (Nox 2) that is engaged during immune activity [46]. Future studies using iPSC derived microglia along with defined activation and inflammatory parameter measures and expanded machine learning tools may increase our ability to predict CBD properties in various contexts.

## Conclusion

Changes in microglial ROS levels can lead to increased extracellular ROS release, which may damage nearby neurons and glial cells through lipid peroxidation and protein oxidation. Monitoring ROS is a crucial strategy for assessing toxicity and inflammation in immune cells such as microglia. In this study, we confirmed the ROS-reducing properties of CBD and evaluated the effects of several inflammatory stimulators, including LPS, Aβ42, and GP120. By combining CellROX imaging with deep learning using CNNs, we developed four novel models capable of classifying microglial images with high accuracy based on their ROS signatures, distinguishing the presence or absence of CBD treatment. Our findings establish a deep learning platform for assessing the effects of CBD on immune cells using ROS-based imaging analysis.

## Supporting information

S1 Fig LPS increases ROS in HMC3 cells. HMC3 microglial cells were treated for 12-hours with 100 ng/ml LPS or vehicle (control) then labeled with CellROX green. Z-stack images obtained using fluorescence confocal microscopy were flattened to create a 3D that was analyzed in ImageJ to determine the ROS signal.
(PDF)

S2 Fig Receiver operator curves (ROC) indicate high model performance. The ROC summarizes the trade-off between the true positive rate and false positive rate. (PDF)
