# Supplementary material for: A predictive machine learning model for cannabinoid effect based on image detection of reactive oxygen species in microglia
Source: PLoS One. 2025 Mar 25;20(3):e0320219. doi: 10.1371/journal.pone.0320219 (PMC11936260; doi:10.1371/journal.pone.0320219)
Supplement: S1 Fig — HMC3 microglial cells were treated for 12-hours with 100 ng/ml LPS or vehicle (control) then labeled with CellROX green. Z-stack images obtained using fluorescence confocal microscopy were flattened to create a 3D that was analyzed in ImageJ to determine the ROS signal. (PDF) [file pone.0320219.s001.pdf]

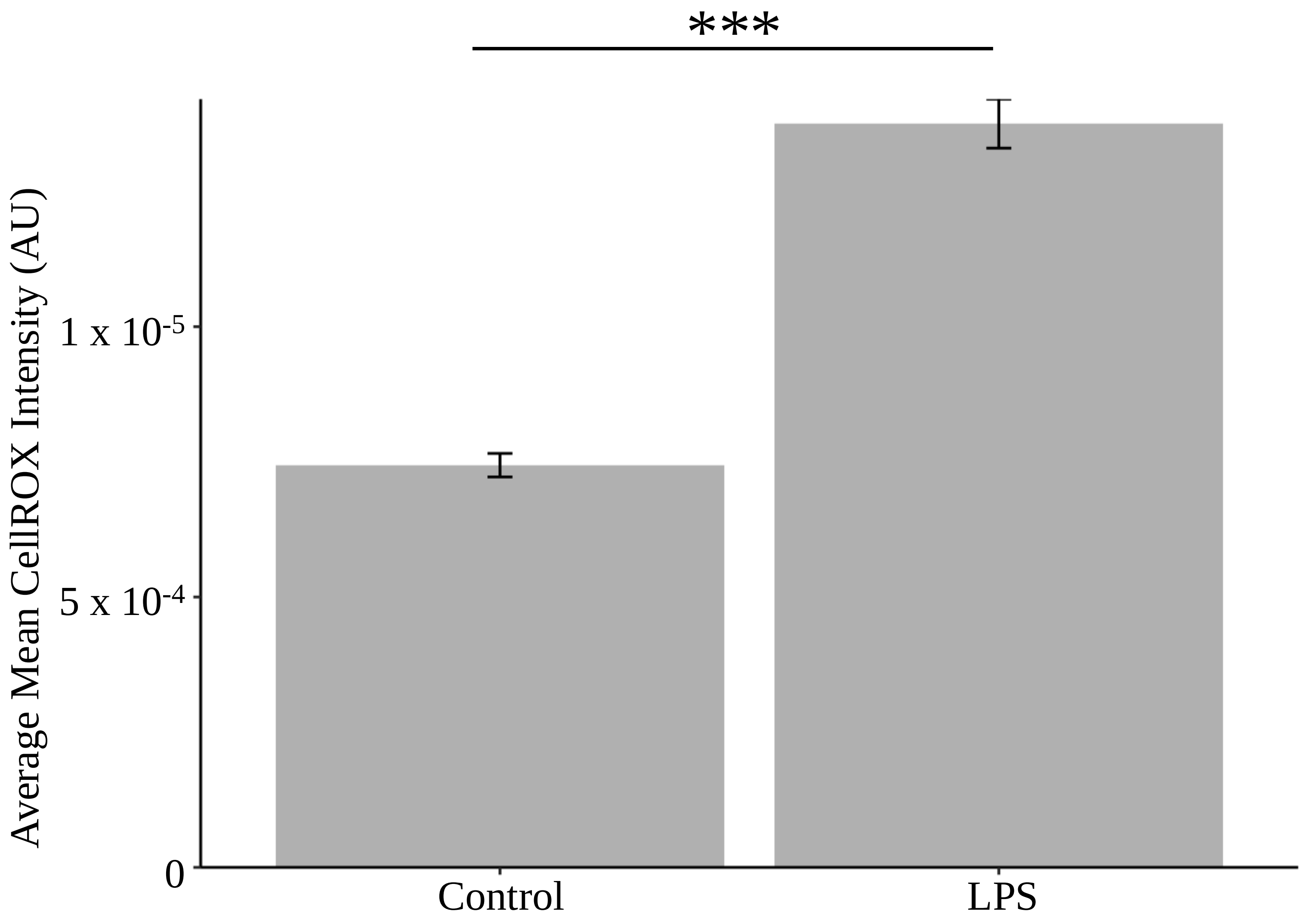

S1 Fig: LPS increases ROS in HMC3 cells. HMC3 microglial cells were treated for 12-hours with 100ng/ml LPS or vehicle (control) then labeled with CellROX green. Z-stack images obtained using fluorescence confocal microscopy were flattened to create a 3D that was analyzed in ImageJ to determine the ROS signal. A Welch's t-test conducted using R statistical software was used to determine statistically significant difference. ***: $p < 2x10^{-16}$
