# Supplementary material for: A predictive machine learning model for cannabinoid effect based on image detection of reactive oxygen species in microglia
Source: PLoS One. 2025 Mar 25;20(3):e0320219. doi: 10.1371/journal.pone.0320219 (PMC11936260; doi:10.1371/journal.pone.0320219)
Supplement: S2 Fig — (PDF) [file pone.0320219.s002.pdf]

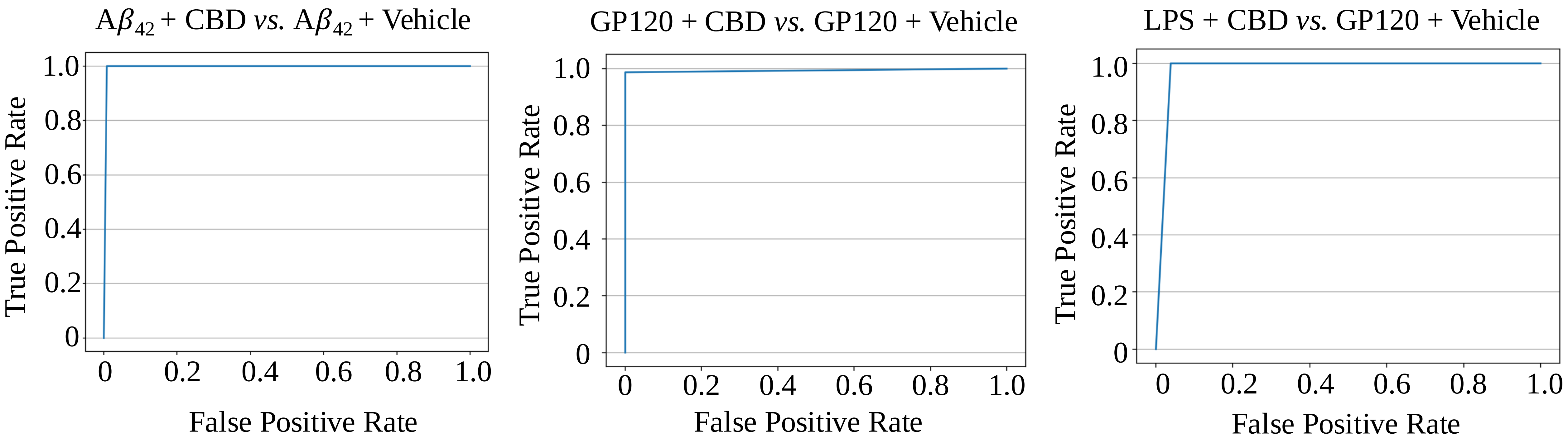

S2 Fig: Receiver operator curves (ROC) indicate high model performance. The ROC summarizes the trade-off between the true positive rate and false positive rate. A score of 1 indicates a perfect classification by the model.
